# Supplementary material for: Enhancing Thermal Stability and Bioaccesibility of Açaí Fruit Polyphenols through Electrohydrodynamic Encapsulation into Zein Electrosprayed Particles
Source: Antioxidants (Basel). 2019 Oct 9;8(10):464. doi: 10.3390/antiox8100464 (PMC6826472; doi:10.3390/antiox8100464)
Supplement: Supplementary file 1 [file antioxidants-08-00464-s001.pdf]

Supplementary Material

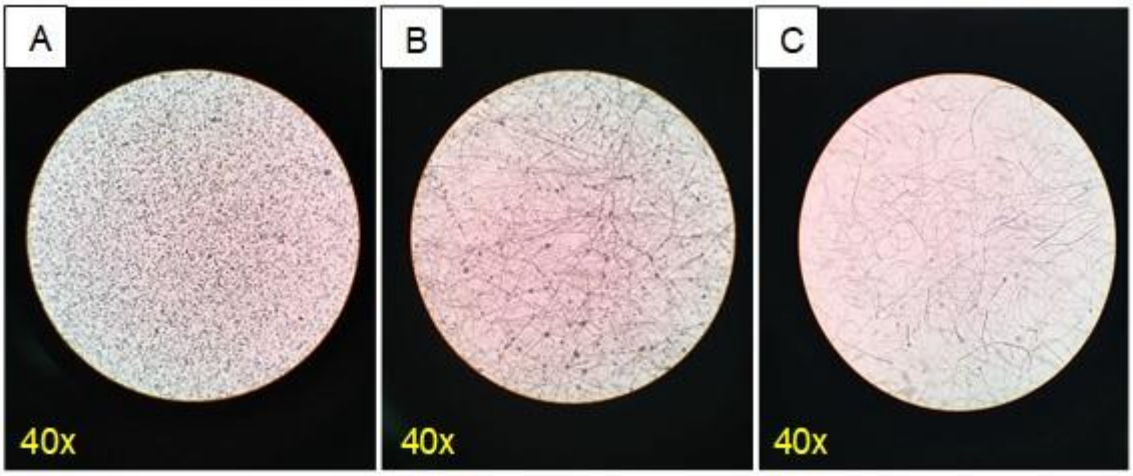

Figure S1. Optical microscope images of electrosprayed samples: A) ZN16-AÇ$_{cc}$; B) ZN18-AÇ$_{cc}$ and C) ZN20-AÇ$_{cc}$.
